# SPG302 protects retinal ganglion cells and preserves visual function by preserving synaptic activity in a mouse model of glaucoma

**DOI:** 10.1016/j.exer.2025.110640

**Published:** 2025-09-16

**Authors:** Tonking Bastola, Seunghwan Choi, Ziyao Shen, Keun-Young Kim, Peter W. Vanderklish, Stella T. Sarraf, Jiun L. Do, Alex S. Huang, Robert N. Weinreb, Won-Kyu Ju

**Affiliations:** a Hamilton Glaucoma Center, and Viterbi Family Department of Ophthalmology and Shiley Eye Institute, University of California San Diego, La Jolla, CA, 92039, USA; b National Center for Microscopy and Imaging Research, Department of Neurosciences, University of California San Diego, La Jolla, CA, 92039, USA; c Spinogenix Inc., 1801 Century Park East, Suite 2400, Los Angeles, CA, 90067, USA; d Institute of Engineering in Medicine, Shu Chien – Gene Lay Department of Bioengineering, University of California San Diego, La Jolla, CA, 92039, USA

**Keywords:** Glaucoma, Retinal ganglion cell, Synapse, SPG302, Visual function

## Abstract

Glaucoma, a leading cause of irreversible vision loss worldwide, is an optic neuropathy characterized by optic nerve degeneration and retinal ganglion cell (RGC) death. Early glaucomatous damage is often associated with dendritic and synaptic abnormalities in RGCs, yet the mechanisms linking these synaptic alterations to RGC death remain unclear. In a mouse model of glaucoma, treatment with the clinical-stage, synaptogenic small molecule SPG302, a pegylated benzothiazole derivative, demonstrated neuroprotective effects, protecting RGCs and their axons in the glaucomatous retina and also improving retinal function as assessed by pattern electroretinogram testing. Elevated intraocular pressure disrupted synapses, as evidenced by reduced synaptophysin expression and homeostatic increases in Bassoon and PSD95 levels in the inner plexiform layer. SPG302 treatment effectively preserved synaptic integrity by reversing these changes. These findings highlight the therapeutic potential of SPG302 for protecting RGCs and preserving vision by modulating synaptic activity in glaucomatous neurodegeneration.

## 1. Introduction

Glaucoma is an optic neuropathy that is a leading cause of irreversible vision loss worldwide. It is characterized by optic nerve degeneration and the loss of retinal ganglion cells (RGC), the principal output neurons of the retina that give rise to axons of the optic nerve (Weinreb et al., 2014, 2016). Currently, glaucoma is treated by reducing intraocular pressure (IOP), the only modifiable risk factor. However, patients can have glaucoma with IOP levels in a normal range (Killer and Pircher, 2018; Leung and Tham, 2022), and glaucoma can progress at such levels as well. Therefore, other factors besides IOP have been hypothesized to also be involved in the degeneration of RGCs. In this case, targeting distinct molecular pathways, such as excitotoxicity, oxidative stress, and mitochondrial dysfunction, may protect RGCs independent of reducing IOP, potentially offering new neuroprotective strategies for glaucoma (Almasieh et al., 2012; Ju et al., 2023; Tezel, 2006).

Dendritic and synaptic abnormalities in RGCs are among the earliest indicators of glaucomatous damage (Bhandari et al., 2019; Morquette and Di Polo, 2008). Elevated IOP leads to various abnormalities in the dendritic compartment of RGCs, including reduced dendritic field radius, shortened total dendritic length, and decreased dendritic branching (Agostinone and Di Polo, 2015; Liu et al., 2011; Morquette and Di Polo, 2008; Weber et al., 1998). Importantly, reductions in the numbers of glutamatergic synapses on RGC dendrites within the inner plexiform layer (IPL) have been observed early during glaucomatous neurodegeneration, prior to the emergence of axonal pathology and the loss of RGC somata (Calkins, 2012; Ou et al., 2016; Park et al., 2014, 2019; Van Hook, 2022). Early changes in synapses may contribute to alterations in RGC excitability and the progression of other degenerative changes in the glaucomatous retina (Agostinone and Di Polo, 2015; Van Hook, 2022). Such observations raise the prospect that synaptic susceptibility may be a critical determinant of glaucoma risk and progression. Accordingly, RGC synapses are metabolically and structurally vulnerable elements that can undergo early retraction or active “pruning” in response to diverse glaucomatous insults, including elevated IOP and cellular stressors that can manifest independently of the level of IOP, such as oxidative stress and inflammation (Agostinone and Di Polo, 2015; Calkins, 2012; Della Santina et al., 2013; Liu et al., 2011; Sun et al., 2023; Van Hook, 2022).

If synaptic degeneration is a key early step in glaucoma pathogenesis, then therapeutics that exhibit synaptic protective or regenerative properties may offer valuable new neuroprotective treatments for glaucoma. Small molecules have been identified that can trigger the rapid genesis of new glutamatergic synapses after transient exposure at low concentrations. Among these, SPG302, a proprietary third-generation compound of the pegylated benzothiazole class and a clinical-stage, orally bioavailable small molecule that targets cytoskeletal signaling pathways involved in synaptogenesis, has been shown to increase glutamatergic synapse density *in vitro* and *in vivo* (Fogarty et al., 2023; Trujillo-Estrada et al., 2021; Zhang et al., 2019). Previous studies have shown that SPG302 and prototype compounds can reverse synaptic and behavioral deficits in animal models of neurodegenerative diseases (Fogarty et al., 2023; Trujillo-Estrada et al., 2021; Zhang et al., 2019). In a mouse model of Alzheimer’s disease (AD), SPG302 rapidly restored hippocampal synaptic density and performance on hippocampus-dependent memory tasks (Trujillo-Estrada et al., 2021). In a highly defined and quantitative model of spinal cord injury that leads to complete unilateral diaphragm paralysis, SPG302 improved the rate and magnitude of recovery of diaphragmatic function (Fogarty et al., 2023), a process thought to involve the enhancement of glutamatergic synaptic input from spared contralateral axons through strengthening of existing synapses, the generation of new synapses, or both (Goshgarian, 2009; Sieck et al., 2021; Sieck and Mantilla, 2009). SPG302 is currently in clinical trials for AD, amyotrophic lateral sclerosis, and schizophrenia, making it well-suited as a candidate synaptic regenerative therapy to consider for glaucoma.

Given the hypothesis that early alterations in synaptic density and function contribute to glaucoma pathogenesis (Della Santina and Ou, 2017; Weber et al., 1998), we investigated whether SPG302 can reverse synapse loss and modify the course of retinal degeneration in a mouse model of glaucoma. Specifically, we evaluated the effects of daily systemic administration of SPG302 on synaptic changes, the integrity of RGCs and their axons within the optic nerve, and visual dysfunction in a mouse model of microbead-induced ocular hypertension.

## 2. Materials and methods

### 2.1. Animals

C57BL/6J mice (The Jackson Laboratory, ME, USA) were housed in covered cages, provided with a standard rodent diet ad libitum, and maintained on a 12 h light/dark cycle. Animals were randomly assigned to experimental and control groups. Visual function tests were conducted using 5-month-old male and female mice. All animal research related to ophthalmic vision adhered to the Association for Research in Vision and Ophthalmology (ARVO) Statement for the Use of Animals in Ophthalmic Vision Research and was performed under protocols approved by the Institutional Animal Care and Use Committee (IACUC) at the University of California, San Diego (CA, USA).

### 2.2. Mouse model of microbead (MB)-induced ocular hypertension

MB-induced ocular hypertension was modeled using a modified protocol based on previous publications (Liu et al., 2020; Sappington et al., 2010). In brief, the eyes of three-month-old C57BL/6J mice were injected with either MB or PBS, as described previously. Anesthesia was induced via intraperitoneal (IP) injection of a ketamine (100 mg/kg, Ketaset; Fort Dodge Animal Health) and xylazine (9 mg/kg, TranquiVed; VEDCO Inc.) cocktail. Topical application of 0.5 % proparacaine hydrochloride was followed by pupil dilation using 1 % tropicamide and 2.5 % phenylephrine. The cornea was then gently punctured with a 30-gauge needle. Subsequently, 2 μl of a 1-μm-diameter polystyrene MB suspension (containing 3.0 × 10^7^ beads), an air bubble, 2 μl of a 6-μm-diameter polystyrene MB suspension (containing 6.3 × 10^6^ beads, Polysciences), and 1 μl of PBS containing 30 % Healon were injected into the anterior chamber using a 32-gauge needle attached to a Hamilton syringe. Then the punctured cornea was sealed using tissue adhesive (3M Vetbond). This procedure induced a moderate elevation in IOP lasting at least 8 weeks. As a control, an equivalent volume of PBS was injected into the contralateral eyes. Eight weeks after MB injection, the mice were sacrificed, and retinal tissues were collected for analysis.

### 2.3. IOP measurement

IOP was measured weekly during IOP elevation using a tonometer (iCare TONOVET, Vantaa, Finland). Contralateral eyes without IOP elevation served as sham controls. Mice were induced and maintained under general anesthesia with 2–4 % Isoflurane in an oxygen-enriched environment (RWD rodent anesthesia machine, TX, USA). IOP was measured in both eyes at room temperature after the mice were placed in the prone position. The tonometer probe was gently placed perpendicular to the central cornea without direct contact and readings were recorded in mmHg. For each mouse, 5 measurements per eye were taken, and the average IOP was used for analysis.

### 2.4. SPG302 administration

SPG302, provided by Spinogenix Inc., was stored at −80 °C until use. The SPG302 stock solution was prepared at a 100X concentration in 100 % DMSO, aliquoted, and stored at −20 °C. During treatment, the stock solution was diluted 1:100 in sterile saline to prepare the injection solution (final DMSO concentration of 1 %). Two groups of 3-month-old C57BL/6J mice were randomly assigned for study following MB injection. One group received vehicle treatment (1 % DMSO in saline, *n* = 20), while the other group was treated with SPG302 (30 mg/kg/day, *n* = 20). The same dose was used in prior studies (Fogarty et al., 2023; Trujillo-Estrada et al., 2021; Zhang et al., 2019). SPG302 or vehicle was administered via a single daily IP injection starting after unilateral MB injection and continuing for 8 weeks.

### 2.5. Tissue preparation

Mice were anesthetized via IP injection of a ketamine (100 mg/kg, Ketaset; Fort Dodge Animal Health, IA, USA) and xylazine (9 mg/kg, TranquiVed; Vedeco, Inc., MO, USA) mixture prior to cervical dislocation. For immunohistochemistry, retinas and ONHs were carefully dissected from the choroids and fixed in 4 % paraformaldehyde (Sigma-Aldrich) prepared in PBS (pH 7.4) for 2 h at 4 °C. Following fixation, retinas and ONHs were washed multiple times with PBS, dehydrated through a graded ethanol series, and embedded in polyester wax.

### 2.6. Whole-mount immunohistochemistry and RGC counting

Retinas from enucleated eyes were dissected as flattened whole-mounts. Retinas were immersed in PBS containing 30 % sucrose for 24 h at 4 °C. The retinas were blocked in PBS containing 3 % donkey serum, 1 % BSA, 1 % fish gelatin, and 0.1 % triton X-100 and incubated with primary antibodies for 3 days at 4 °C. The primary antibody used was rabbit polyclonal anti-RBPMS antibody. After several wash steps, the retinas were incubated with Alexa Fluor-488 conjugated donkey anti-rabbit IgG antibody or Alexa Fluor-568 conjugated donkey anti-rabbit IgG antibody for 24 h, and subsequently washed with PBS. Images were acquired with Keyence All-in-One Fluorescence microscopy (BZ-X810, Keyence Corp. of America). RNA-binding protein with multiple splicing (RBPMS)- or Neuron-specific class III beta-tubulin (TUJ1)-positive RGCs were counted in two zones by middle and/or peripheral retina (three-sixths and five-sixths of the retinal radius), and the scores were averaged using ImageJ software [National Institutes of Health (NIH), MD, USA].

### 2.7. Immunohistochemistry

Immunohistochemical staining was performed on 7-μm wax sections of full-thickness retina and glial lamina. Sections from wax blocks (*n* = 3 mice per group) were used for analysis. To minimize non-specific background staining, tissues were incubated in 1 % bovine serum albumin (BSA, Sigma-Aldrich) in PBS for 1 h at room temperature, followed by incubation with primary antibodies for 16 h at 4 °C. Primary antibody used were mouse monoclonal anti-neurofilament 68 (NF68) antibody, rabbit polyclonal anti-synaptophysin antibody, mouse monoclonal anti-TUJ1 antibody, mouse monoclonal anti-bassoon antibody, and rabbit polyclonal anti-postsynaptic density protein-95 (PSD95) antibody. After several washes with PBS, tissue sections were incubated with the secondary antibodies, Alexa Fluor-488 conjugated donkey anti-rabbit IgG antibody or Alexa Fluor-568 conjugated donkey anti-mouse IgG antibody, for 4 h at 4 °C and washed them again. The sections were counterstained with Hoechst 33342 (1 μg/ml; Invitrogen, CA, USA) in PBS. Images were captured using Keyence All-in-One Fluorescence Microscopy (BZ-X810, Keyence Corp. of America, IL, USA). The fluorescent integrated intensity of each target protein, expressed as pixels per area, was quantified using ImageJ software (NIH), with imaging parameters kept constant and background subtraction applied. The primary and secondary antibodies used are detailed in Supplementary Table 1.

### 2.8. Transmission electron microscopy analysis

Optic nerve tissues were fixed through cardiac perfusion at 37 °C using a solution containing 2 % paraformaldehyde and 2.5 % glutaraldehyde (Ted Pella) in 0.15 M sodium cacodylate buffer (pH 7.4). The tissues were then placed in precooled fixative on ice for 1 h, as described previously [9, 29, 32]. The ONH tissues were embedded in Durcupan ACM resin (Fluka, St. Louis, MO, USA). Ultrathin sections (70 nm) were post-stained with uranyl acetate and lead salts and examined using a JEOL 1200FX transmission electron microscope (EM) operated at 80 kV (Tokyo, Japan). Images were captured on film at 8000 × magnification. For quantitative analysis, axon counts were performed using ImageJ software (NIH).

### 2.9. Pattern electroretinogram (pERG) and pattern visual evoked potential (pVEP) analysis

pERG and pVEP were measured using the Celeris apparatus (Diagnosys, MA, USA), as described previously (Choi et al., 2020; Ju et al., 2025). After overnight dark adaptation, mice were anesthetized under red light using an intraperitoneal injection of a ketamine/xylazine cocktail. To dilate the pupils, a mixture of 0.5 % tropicamide and 2.5 % phenylephrine was applied directly to the eyes. Additionally, 1 % proparacaine drops and 0.3 % hypromellose gel were used to prevent corneal drying and cataract formation. pERG responses were recorded using alternating black-and-white vertical stimuli reversing at 1 Hz (2 reversals per second) with a luminance of 50 cd/m^2^, delivered via a pattern stimulator. For each eye, 200 traces were recorded, and averaged waveforms were calculated by measuring amplitudes (μV) from the P1 peak to the N2 trough. Simultaneously, pVEP responses were captured. For pVEP recordings, a ground electrode was positioned subcutaneously at the tail, a reference electrode at the snout, and an active electrode subdermally at the midline of the head over the visual cortex. Each eye received 100 flashes of 1 Hz, 0.05 cd s/m^2^ white light through corneal stimulators, with responses recorded over 300 ms at a sampling frequency of 2000 Hz. A total of 200 traces per eye were recorded, and averaged waveforms were calculated by measuring amplitudes (μV) from the P1 peak to the N1 trough. For each mouse, five trials were performed, with 100 sweeps per trial. The filter cutoffs for pVEP recordings were set to 1.25 Hz (low) and 100 Hz (high). All recordings were conducted while maintaining the body temperature between 37 °C and 38 °C using system heat pads. Data analysis was performed using Espion V6 software (Diagnosys) (Gramlich et al., 2021; Queirós et al., 2020).

### 2.10. Statistical analysis

Data are shown as the mean standard error of the mean (SEM). Depending on the experimental design, statistical multiple-group comparison or one-way ANOVA was applied. All statistical analyses were conducted using GraphPad Prism software (GraphPad, CA, USA), with significance defined as a P-value less than 0.05.

## 3. Results

### 3.1. SPG302 administration protects RGCs in glaucomatous mice

**Fig. 1.**
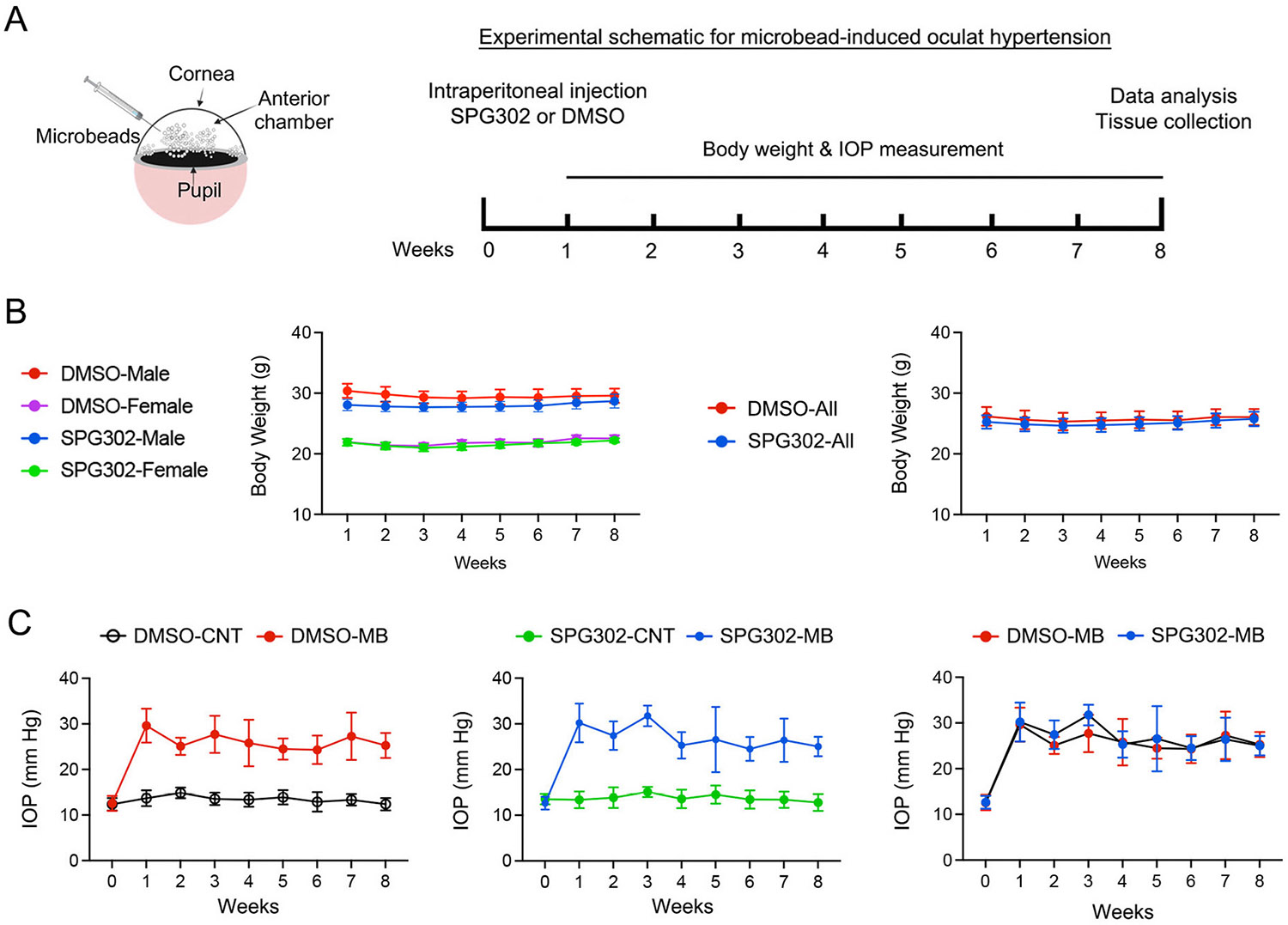
Glaucoma modeling with MB injection and IOP elevation. (A) Experimental schematic and timeline of MB injection, SPG302 administration, body weight and IOP measurements, and tissue collection and data analysis in mice. (B) Body weight measurement (*n* = 10 mice per group). (C) IOP measurement (*n* = 10 mice per group). Error bars represent SEM.

**Fig. 2.**
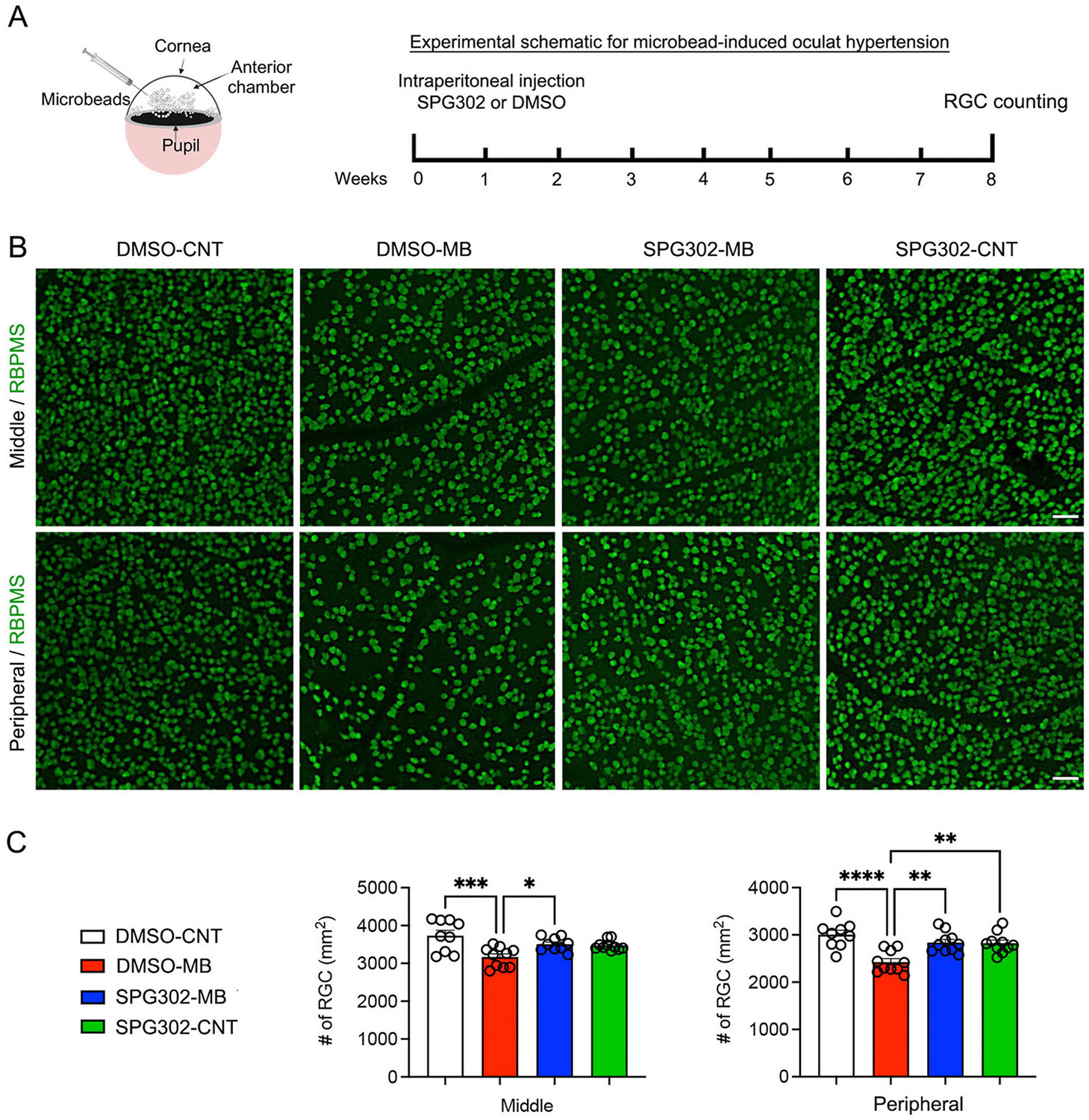
SPG302 administration promotes RGC survival in glaucomatous mice. (A) Experimental schematic and timeline of MB injection, SPG302 administration, and RGC counting in mice. (B) Representative retina wholemount images for RBPMS (green)-positive RGCs in the middle and peripheral retina. (C) Quantitative analysis of RGC numbers in the middle and peripheral areas of the retina (*n* = 9 or 10 mice per group). Error bars represent SEM. Statistical analysis was performed using one-way ANOVA and Tukey’s multiple comparisons test. *P < 0.05, **P < 0.01, ***P < 0.001 and ****P < 0.0001. Scale bars, 50 μm.

Given the potential early and causative role of synapse loss in glaucoma, we investigated the neuroprotective effects of the synaptic regenerative small molecule, SPG302 (Trujillo-Estrada et al., 2021), on retinal ganglion cell (RGC) survival and axonal preservation in a microbead (MB)-induced ocular hypertension mouse model of glaucoma. Three-month-old C57BL/6J mice were treated daily with intraperitoneal SPG302 for eight weeks following MB injection (Fig. 1A). Body weight and IOP were monitored weekly after MB and SPG302 injection, and RGC counts were assessed eight weeks post-injection (Fig. 1A-C). There were no statistically significant differences in body weight between DMSO and SPG302 administration (Fig. 1B). To reduce variability, mice with IOPs ranging from 20 to 30 mmHg were selected for analysis (Fig. 1C). There was no significant difference in IOP levels between DMSO-treated MB (DMSO-MB) mice and SPG302-treated MB (SPG302-MB) mice (Fig. 1C). In RGC counting analysis, MB-induced IOP elevation in DMSO-MB mice resulted in a significant reduction in RGC numbers in the middle and peripheral areas in the retina compared to DMSO-control (DMSO-CNT) mice (Fig. 2A-C; Supplementary Table 2). Importantly, SPG302 treatment markedly enhanced RGC survival in the middle and peripheral areas in the retina of MB-injected mice (Fig. 2A-C; Supplementary Table 2). Additionally, there was no significant difference in RGC numbers between DMSO-CNT mice and SPG302-CNT mice (Fig. 2C; Supplementary Table 2).

### 3.2. SPG302 administration preserves RGC axons in glaucomatous mice

**Fig. 3.**
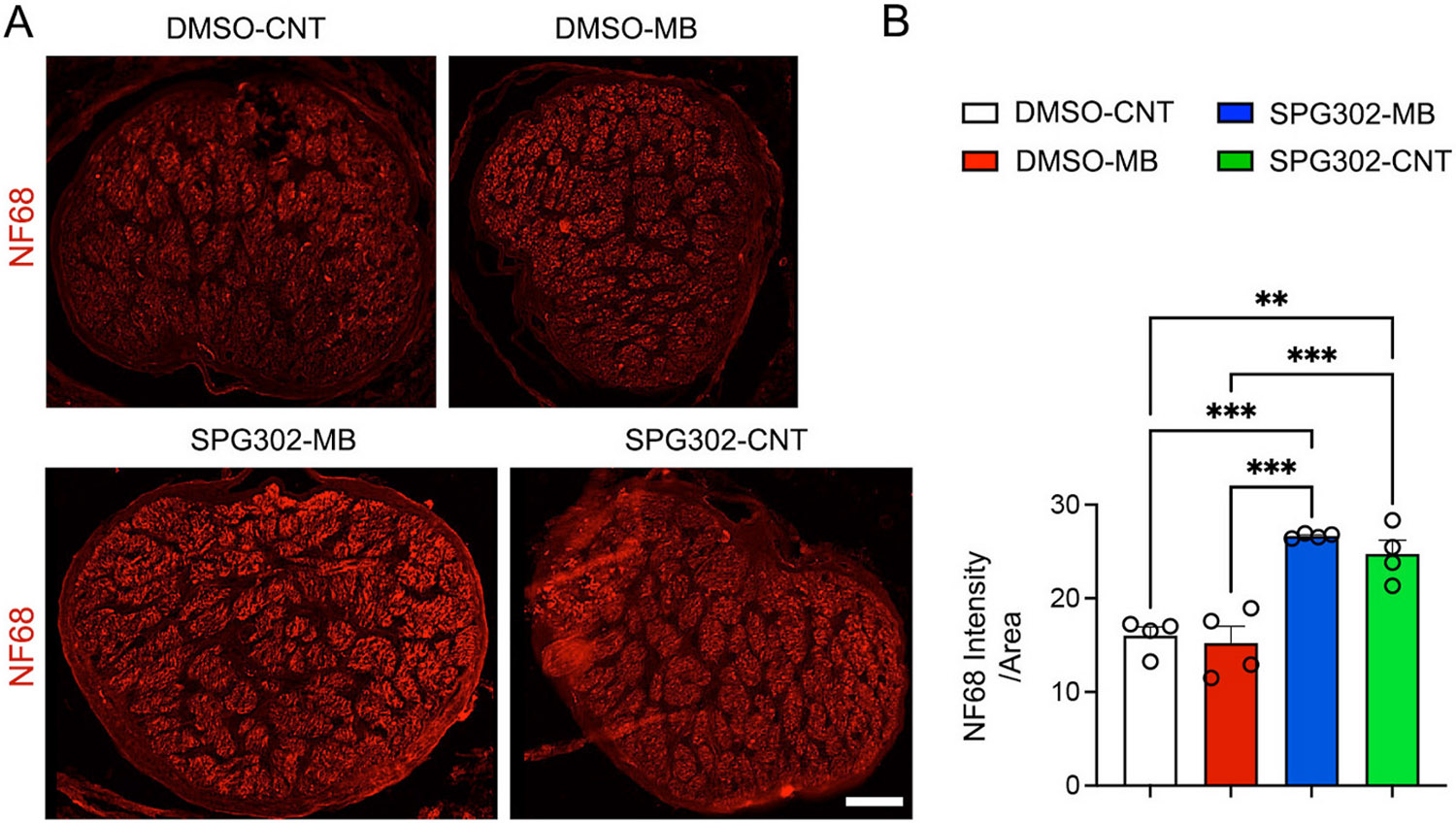
SPG302 administration enhances NF68 expression in RGC axons in the glial lamina of glaucomatous mice. (A) Representative images from the glial lamina for NF68 (green)-positive axons. (B) Quantitative analysis of NF68 intensity in the glial lamina (*n* = 4 mice per group). Error bars represent SEM. Statistical analysis was performed using one-way ANOVA and Tukey’s multiple comparisons test. **P < 0.01 and ***P < 0.001. Scale bars, 50 μm.

Based on our observation of SPG302-mediated RGC protection, we investigated whether SPG302 administration protects RGC axons. We performed immunohistochemistry using neurofilament 68 (NF68) as an axonal marker. MB-induced elevated IOP did not alter the NF68 intensity in the glial lamina compared to DMSO-CNT (Fig. 3A and B). Of note, SPG302 administration significantly increased NF68 immunoreactivity in the glial lamina of glaucomatous MB mice compared to DMSO-MB mice (Fig. 3A and B). Additionally, SPG302-CNT mice displayed markedly elevated NF68 immunoreactivity in the glial lamina relative to DMSO-CNT and DMSO-MB groups (Fig. 3A and B). NF68 levels did not decrease in MB-injected eyes, likely because the mild to moderate stress induced by MB was insufficient to cause detectable neurofilament loss within the experimental timeframe. The increased NF68 observed in the SPG302-treated group may indicate enhanced axonal stability or compensatory cytoskeletal remodeling induced by the synaptogenic treatment. Consequently, no significant difference was detected between CNT and MB-injected treated eyes, consistent with SPG302’s preservation of axonal integrity.

**Fig. 4.**
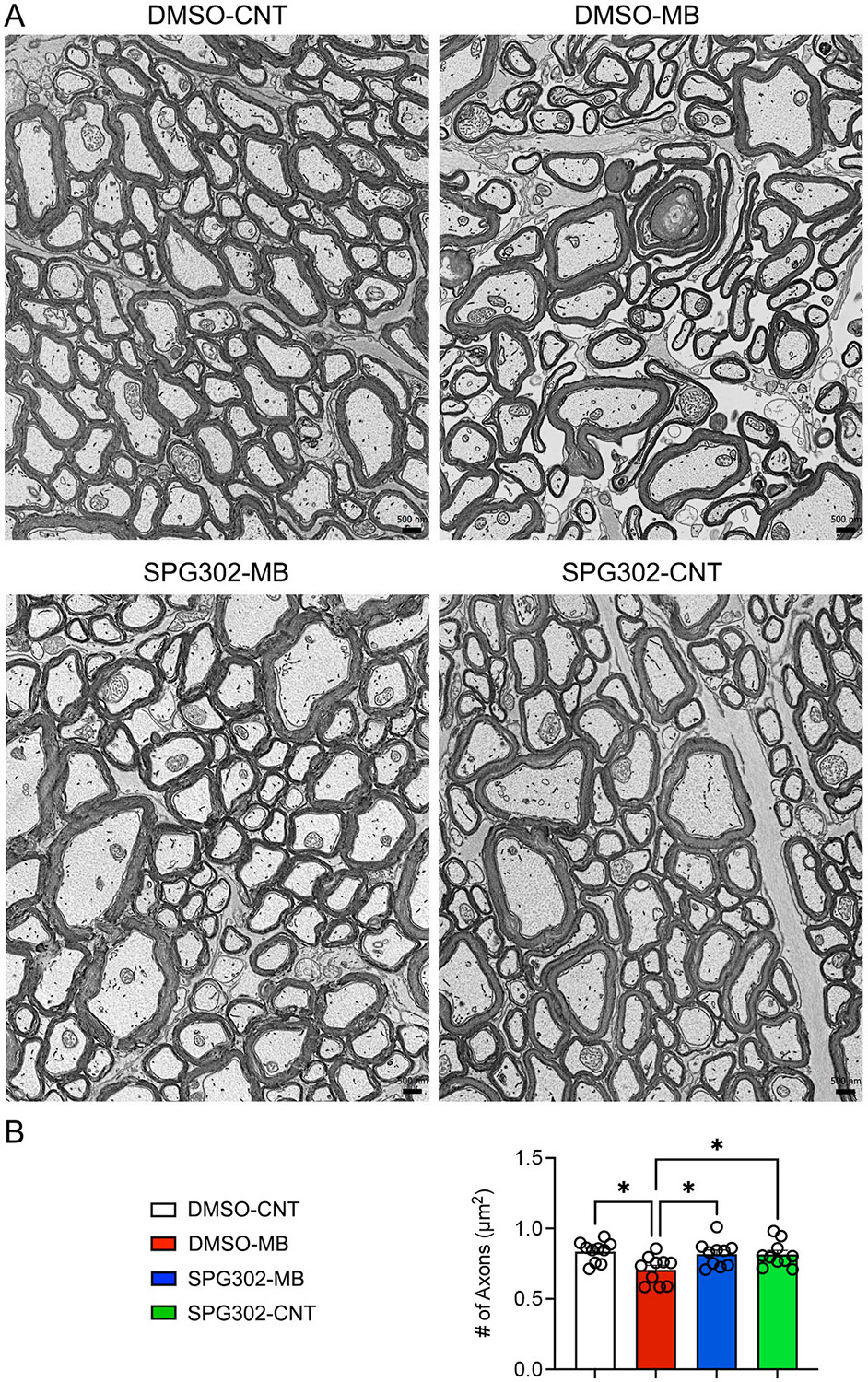
SPG302 administration protects RGC axons in the ON of glaucomatous mice. (A) Representative images for axons with myelination in the ON. (B) Quantitative analysis of axon numbers in the ON (*n* = 10 images from 5 mice per group). Error bars represent SEM. Statistical analysis was performed using one-way ANOVA and Tukey’s multiple comparisons test. *P < 0.05. Scale bars, 500 nm.

To further assess the role of SPG302 in preserving axonal integrity during glaucomatous neurodegeneration, we analyzed RGC axon counts and myelination morphology in the optic nerve (ON) beyond the myelination transition zone (MTZ) using transmission electron microscopy (TEM). Compared to DMSO-CNT mice, DMSO-MB mice exhibited a marked reduction in axon numbers and disrupted myelination in the ON (Fig. 4A). However, SPG302 administration preserved both axonal structure and myelination in the ON of glaucomatous MB mice (Fig. 4A). Quantitative two-dimensional electron microscopy analysis further revealed that elevated IOP significantly decreased axon numbers in the ON of DMSO-MB mice compared to DMSO-CNT controls (Fig. 4B; Supplementary Table 3). Notably, SPG302 treatment significantly restored axon numbers and preserved myelination in the ON of glaucomatous MB mice (Fig. 4B; Supplementary Table 3).

### 3.3. SPG302 administration ameliorates visual dysfunction in glaucomatous mice

**Fig. 5.**
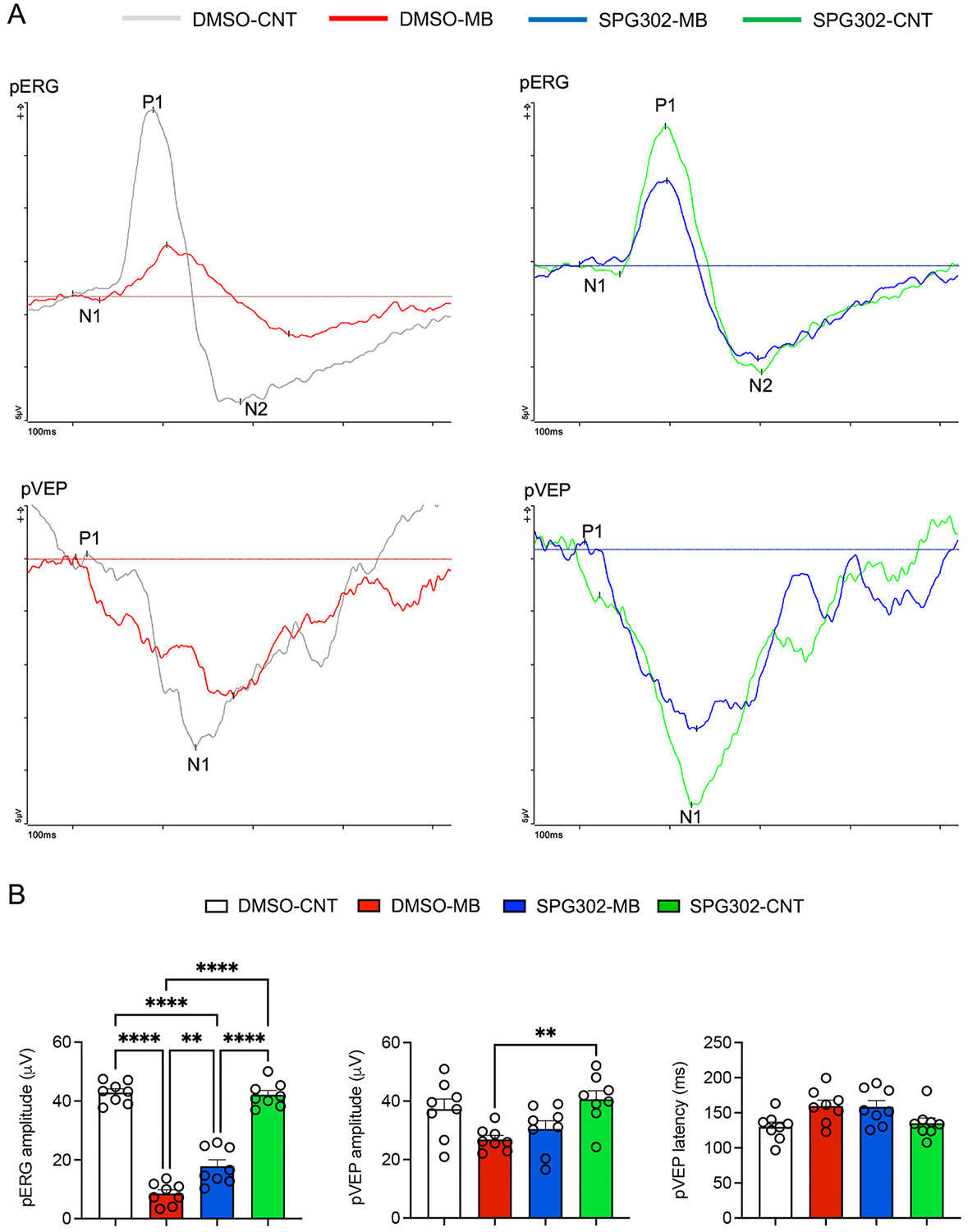
SPG302 administration ameliorates visual dysfunction in glaucomatous mice. (A) Representative recording graphs of pERG and pVEP tests. (B) Quantitative analysis of pERG amplitudes and pVEP amplitude and latency (*n* = 8 mice per group). Error bars represent SEM. Statistical analysis was performed using one-way ANOVA and Tukey’s multiple comparisons test. **P < 0.01 and ****P < 0.0001.

To evaluate the effects of SPG302 administration on visual function in the MB model, we assessed both pattern electroretinogram (pERG) and pattern visual evoked potential (pVEP) metrics. MB-induced IOP elevation caused a significant reduction in RGC function, as evidenced by a decrease in pERG amplitude in DMSO-MB mice compared to DMSO-CNT controls (Fig. 5A and B; Supplementary Table 4). However, no significant differences were observed in the number of functional ON fibers or conduction delay, as indicated by pVEP amplitude and latency, respectively, following MB-induced IOP elevation (Fig. 5A and B; Supplementary Table 4). Notably, SPG302 administration significantly improved RGC function in MB-injected mice as evidenced by increased pERG amplitude compared with DMSO-MB, though no statistical differences were detected in pVEP amplitude or latency (Fig. 5A and B; Supplementary Table 4).

### 3.4. SPG302 administration preserves synaptic activity in the retina in glaucomatous MB mice

The reported synaptic regenerative effects of SPG302 are accompanied by changes in postsynaptic proteins such as PSD95 and the colocalization of these proteins with presynaptic markers, an indicator of functional synapses (Trujillo-Estrada et al., 2021). We thus studied the impact of SPG302 administration on synaptic markers in the MB model. To evaluate the changes in presynaptic and postsynaptic activities in the retina of MB-injected mice, we performed immunohistochemical analysis using antibodies for synaptophysin, a marker for presynaptic protein in the synaptic vesicles, TUJ1, a marker for RGCs (soma and neurites), Bassoon, a marker for presynaptic protein in the active zone of synapses, and PSD95, a postsynaptic density scaffolding protein that marks postsynaptic elements.

**Fig. 6.**
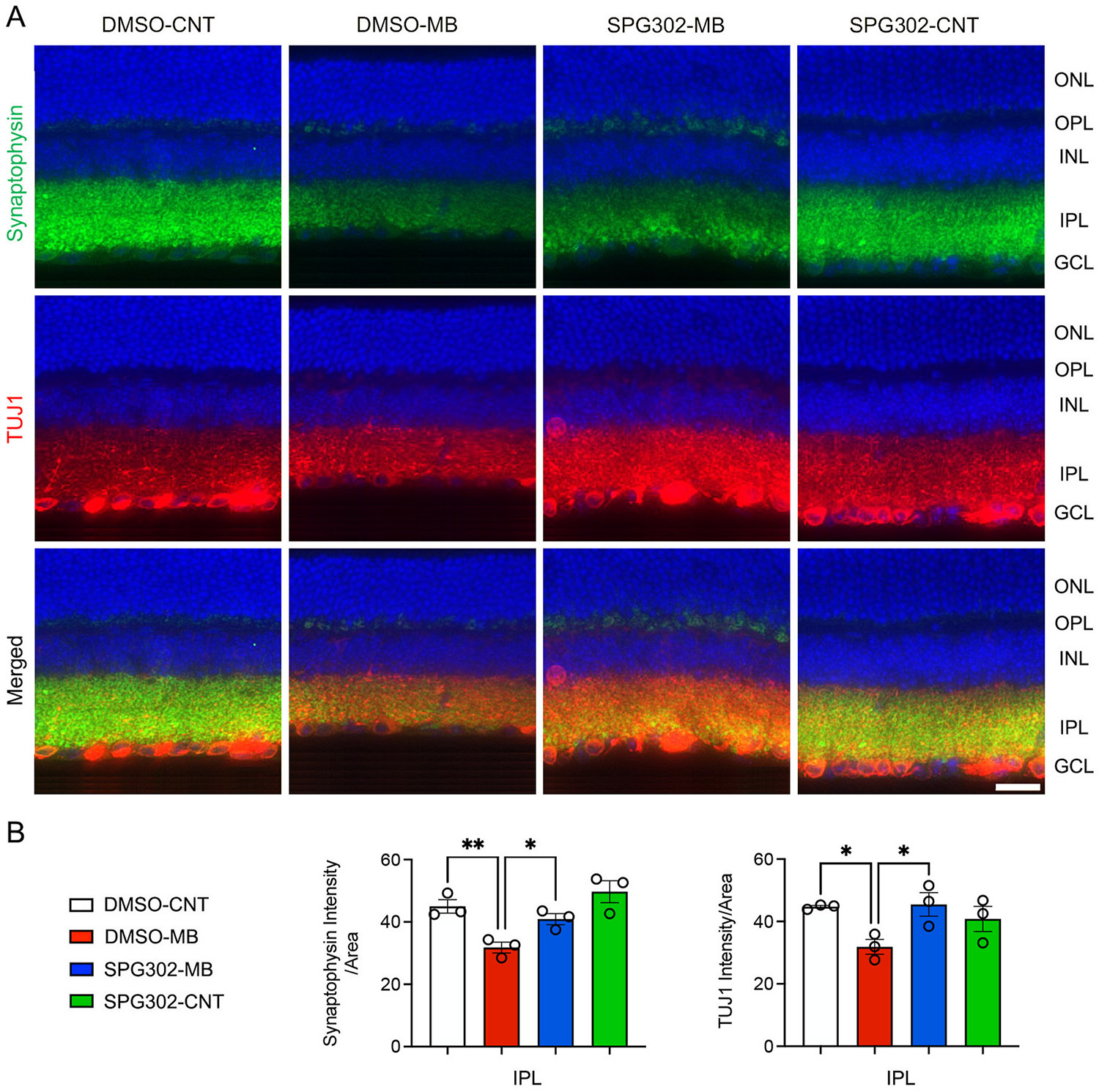
SPG302 administration preserves synaptophysin protein expression in glaucomatous mice. (A) Representative retinal section images for synaptophysin (green) and TUJ1 (red) protein expression. (B) Quantitative analysis of synaptophysin and TUJ1 intensity in the retina (*n* = 4 mice per group). Error bars represent SEM. Statistical analysis was performed using one-way ANOVA and Tukey’s multiple comparisons test. *P < 0.05 and *P < 0.01. Scale bars, 20 μm.

**Fig. 7.**
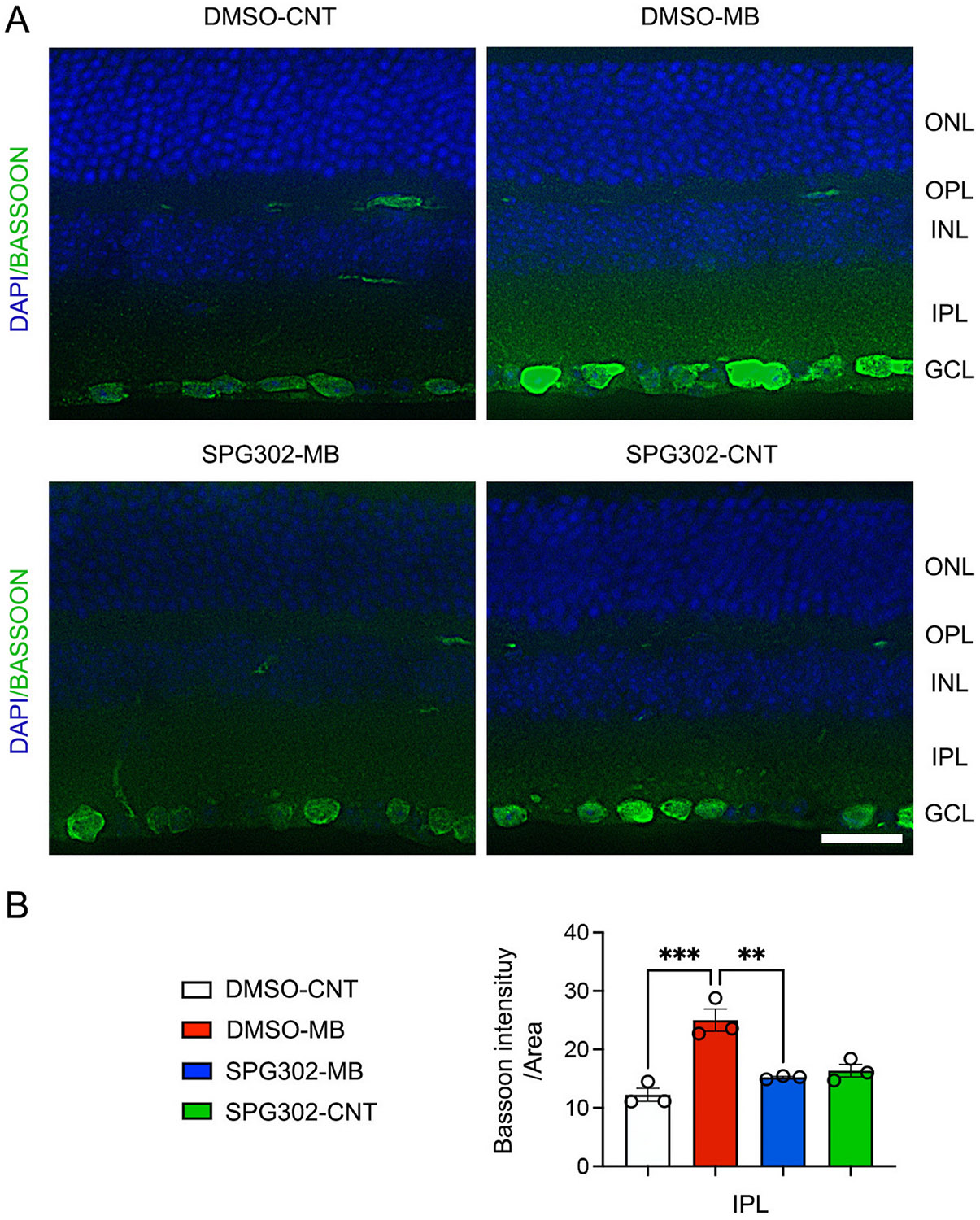
SPG302 administration preserves bassoon protein expression in glaucomatous mice. (A) Representative retinal section images for bassoon (green) protein expression. (B) Quantitative analysis of bassoon intensity in the retina (*n* = 4 mice per group). Error bars represent SEM. Statistical analysis was performed using one-way ANOVA and Tukey’s multiple comparisons test. **P < 0.01 and ***P < 0.001. Scale bars, 20 μm.

**Fig. 8.**
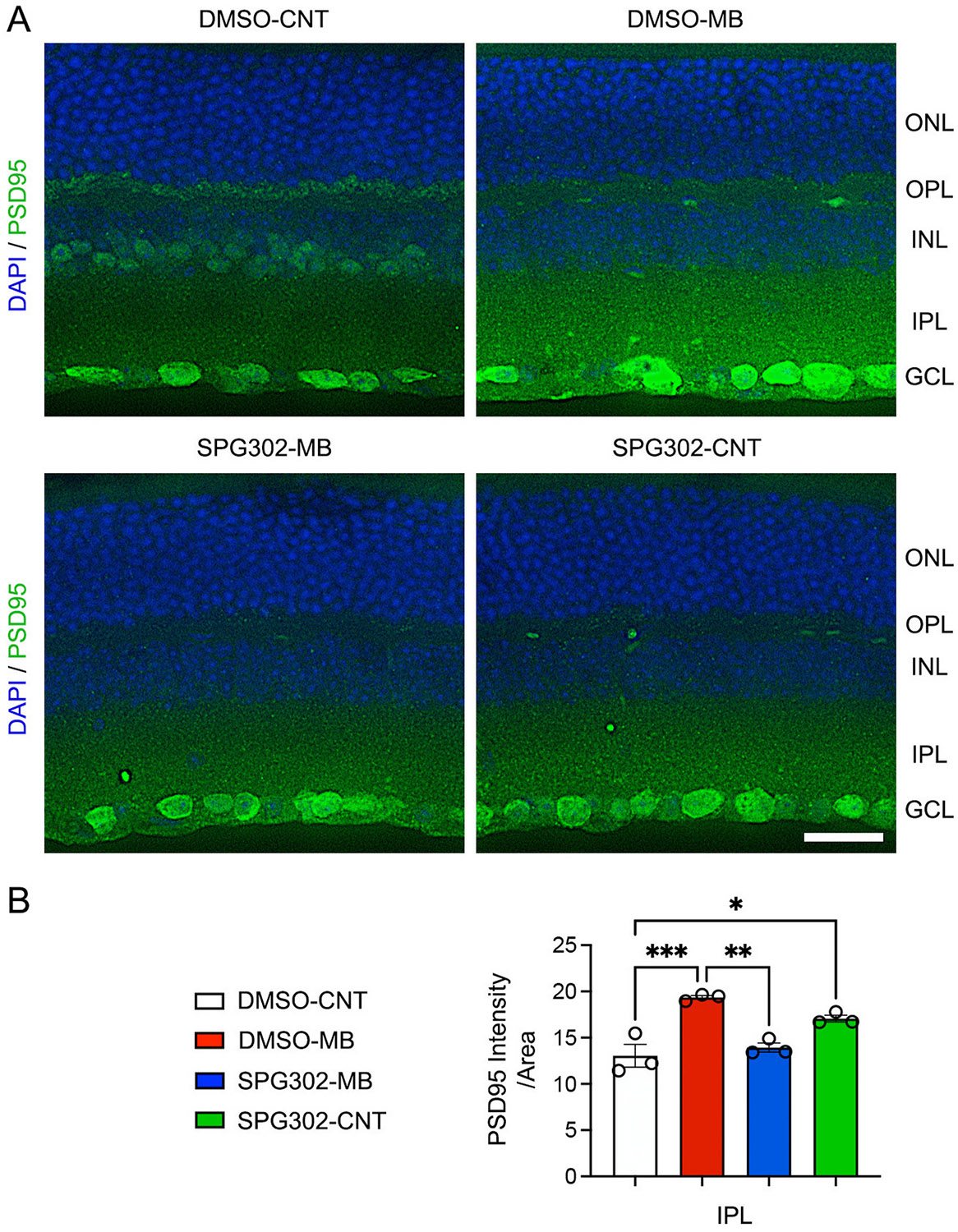
SPG302 administration preserves PSD95 protein expression in glaucomatous mice. (A) Representative retinal section images for PSD95 (green) protein expression. (B) Quantitative analysis of PSD95 intensity in the retina (*n* = 4 mice per group). Error bars represent SEM. Statistical analysis was performed using one-way ANOVA and Tukey’s multiple comparisons test. *P < 0.05, **P < 0.01, and ***P < 0.001. Scale bars, 20 μm.

MB-induced elevated IOP reduced synaptophysin intensity in the IPL compared to the DMSO-CNT retina (Fig. 6A), along with the reduction of TUJ1 intensity. In contrast, SPG302 administration restored the synaptophysin and TUJ1 intensities in the IPL of MB-treated mice compared to the DMSO-MB retina (Fig. 6A). Quantitative analysis confirmed a significant restoration of synaptophysin and TUJ1 intensities to near control levels in the IPL of SPG302-MB compared to the DMSO-MB retina (Fig. 6B). Additionally, there were no significant differences in the synaptophysin and TUJ1 intensities between DMSO-CNT mice and SPG302-CNT mice (Fig. 6B). Consistent with prior reports (Hernandez et al., 2009; Park et al., 2014), MB-induced elevated IOP increased the labeling of Bassoon and PSD95 in the IPL and ganglion cell layer (GCL), a change thought to reflect homeostatic responses to synaptic compromise. Bassoon intensity in the IPL and GCL was elevated in DMSO-MB retina compared to the DMSO-CNT retina (Fig. 7A), and SPG302 administration restored the Bassoon intensity in the IPL and GCL compared to DMSO-MB retina (Fig. 7A). Quantitative analysis confirmed a significant restoration of Bassoon intensity in the IPL of SPG302-MB compared to the DMSO-MB retina (Fig. 7B). Additionally, there was no significant difference in the Bassoon intensity between DMSO-CNT mice and SPG302-CNT mice (Fig. 7B). MB-induced elevated IOP also increased PSD95 intensity in the IPL and GCL compared to the DMSO-CNT retina (Fig. 8A). As with its effects on Bassoon, SPG302 administration restored the PSD95 intensity in the IPL and GCL compared to the DMSO-MB retina (Fig. 8A). Quantitative analysis confirmed a significant restoration of PSD95 intensity in the IPL of SPG302-MB compared to the DMSO-MB retina (Fig. 8B). Interestingly, SPG302-CNT showed a considerable increase of PSD95 intensity compared to the DMSO-CNT (Fig. 8B).

## 4. Discussion

The findings described herein support the hypothesis that drugs that promote synaptic regeneration potentially offer a viable new neuroprotective strategy in glaucoma. In addition, they highlight the potential therapeutic utility of the clinical-stage drug SPG302 in glaucoma. SPG302 treatment in mice exhibiting microbead-induced elevations in IOP was efficacious in preserving RGCs, their axons, and visual function in the context of glaucomatous neurodegeneration. Notably, this protective effect was associated with the preservation of synapses as evidenced by normalization of the expression levels of pre- and post-synaptic proteins, including synaptophysin, Bassoon, and PSD95, as well as TUJ1, which spans the soma, dendrites, and axons (Bastola et al., 2024; Zhang et al., 2010).

SPG302 and its derivatives represent a new class of potent, rapid-acting synaptic regenerative small molecules and have been widely tested in animal models of various neurologic diseases, showing the ability to regenerate dendritic spine synapses and improve neurological function (Fogarty et al., 2023; Trujillo-Estrada et al., 2021; Zhang et al., 2019). In the triple transgenic mouse model of Alzheimer’s disease, daily treatment with SPG302 reversed large deficits in dendritic spine density in the hippocampus, returning synaptic density to that observed in wild-type mice, and rescued performance in two hippocampus-dependent memory tasks (Trujillo-Estrada et al., 2021). SPG302 has also been shown to improve respiratory motor function in a rat model of spinal cord injury (SCI) (Fogarty et al., 2023) wherein complete unilateral loss of diaphragm function is induced by hemisection at the level of C2. Partial recovery can occur in this model, which is thought to be mediated by synaptogenesis between spared contralateral axons and phrenic motor neurons. SPG302 greatly improved the rate and magnitude of functional diaphragmatic recovery as assessed by diaphragmatic electromyography. These and other findings have prompted the initiation of clinical trials of SPG302 as a once-a-day orally bioavailable synaptic regenerative therapy in multiple indications that have been shown to involve the loss of axospinous glutamatergic synapses: amyotrophic lateral sclerosis (NCT05882695), Alzheimer’s disease (NCT06427668), and schizophrenia (NCT06442462). In this context, and considering the studies reported here, it is reasonable to consider SPG302 as a candidate therapy for clinical evaluation in glaucoma.

Glaucoma, a neurodegenerative disease of the retina, is associated with early changes in the synaptodendritic compartment of RGCs, including alterations in glutamatergic synapses that multiple investigators have suggested are one of the earliest signs of disease (Agostinone and Di Polo, 2015; Hsieh et al., 2006; Liu et al., 2011; Shou et al., 2003; Tsai et al., 2004; Weber and Harman, 2005; Weber et al., 1998). Observations that synaptic compromise in the retina precedes morphological alterations in dendrites, somata, and axons point to synaptic vulnerability as a key early determinant of glaucoma onset and progression. Whether RGC dendritic loss is a primary insult or secondarily triggered by axon injury is an active area of research, although some data support that axonal injury triggers rapid structure alteration in RGC dendritic arbors before the manifest axon loss (Agostinone and Di Polo, 2015). This possibility finds support in parallels between glaucoma and AD, a neurodegenerative disease widely regarded as a synaptic failure. Rates of glaucoma are roughly two- to five-fold higher in AD patients, and there is a large increase in the rate of glaucomatous degeneration in AD patients who have elevated IOP compared to the rate in cognitively normal individuals with elevated IOP (Sen et al., 2020). Such observations suggest that the underlying pathology in glutamatergic synapses predisposes to glaucoma. Importantly, while synaptic compromise may be a convergence point between pathophysiologic mechanisms contributing to glaucoma, glaucomatous changes in synaptodendritic and axonal compartments, including early synapse loss, are manifest in multiple animal models of elevated IOP (Berry et al., 2015; Park et al., 2014), indicating that such IOP is sufficient to trigger such changes.

In the microbead glaucoma model, we detected marked alterations in pre- and postsynaptic components within the retina, as evidenced by reductions in synaptophysin and TUJ1, and increases in Bassoon and PSD95 expression, in the IPL, indicating compromise of pre- and post-synaptic elements and potentially dendritic integrity. Remarkably, the administration of SPG302 effectively reversed both of these changes. Synaptophysin, a synaptic vesicle protein critical for vesicle trafficking, fusion, and recycling, has a key role in neurotransmitter release in the central nervous system, including the retina (Kapfhammer et al., 1994; Wiedenmann and Franke, 1985; Yang et al., 2002). At the same time, Bassoon, a presynaptic protein localized in the active zone, anchors synaptic vesicles and organizes the neurotransmitter release machinery in the retina (Dick et al., 2003; Zanazzi and Matthews, 2009). Our findings suggest that reduced synaptophysin may reflect impaired vesicle dynamics, potentially disrupting neurotransmitter release in the glaucomatous retina. In contrast, increased Bassoon levels may occur during elevated IOP. They may indicate a potential homeostatic response to compensate for synaptic loss or damage, or represent plastic changes in response to glaucomatous injury (Hernandez et al., 2009). Thus, we proposed that SPG302 preserves presynaptic activity during elevated IOP, normalizing vesicle dynamics and preventing both synaptic loss and homeostatic responses to glaucomatous insults such as elevated IOP.

PSD95, a post-synaptic scaffold protein that anchors and organizes glutamate receptors (particularly NMDA receptors) at excitatory synapses, is predominantly localized to the OPL and IPL in the retina, where photoreceptors, bipolar cells, and RGCs form synaptic connections (Fletcher et al., 2000; Koulen et al., 1998). In this study, we observed a significant increase in PSD95 expression in the IPL of the glaucomatous retina after microbead-induced ocular hypertension, a change that was reversed by treatment with SPG302. Our finding of elevated PSD95 in MB-treated animals replicates results using episcleral vein cautery-induced ocular hypertension (Park et al., 2014), but is the opposite of that seen with episcleral vein laser-induced ocular hypertension (Ou et al., 2016). Differences may be due to the methods and models used in evaluating native PSD95 in the present study (Park et al., 2014) vs. transfected PSD95 (Ou et al., 2016). Another possibility is that, since our model exhibits mild damage characterized by less than 20 % RGC death, upregulation of PSD95 is a compensatory or protective mechanism aiming to strengthen or preserve excitatory synaptic connections in RGCs in response to mild glaucomatous injury.

In the present study, SPG302 administration significantly improved RGC survival and axonal integrity while preserving visual function, as evidenced by sustained pERG amplitudes in response to elevated IOP. Because pERG primarily reflects RGC activity (Holder, 2001), a decline in its amplitude signals the onset of early or localized RGC impairment. Hence, the pERG reduction observed under elevated IOP likely indicates that RGCs are affected before extensive axonal damage becomes evident. In contrast, pVEP measures cortical responses to visual input (Holder, 2001) and incorporates the entire visual pathway from the retina to the visual cortex. It typically remains unaffected in mild or moderate RGC dysfunction, manifesting changes only after substantial axonal damage. Therefore, a decreased pERG coupled with normal pVEP indicates early or moderate RGC dysfunction without significant conduction deficits detectable at the cortical level. These findings align with the moderate RGC loss observed in our MB model following IOP elevation. They are characteristic of early-stage glaucoma, wherein retinal-level damage precedes marked alterations along the optic pathway. Thus, the effects of SPG302 in partially reversing pERG functional changes support its potential for therapeutic utility in early to moderate glaucoma.

A potential limitation of this study is that only a single dose of SPG302 (30 mg/kg) was evaluated, chosen based on prior animal research (Fogarty et al., 2023; Trujillo-Estrada et al., 2021) that demonstrated improved structural dendritic integrity and neuronal function. While SPG302 is readily brain penetrant, the efficiency of drug delivery to the eye may differ. Therefore, exploring additional doses and establishing a dose-dependent response would enhance the biological plausibility and robustness of these findings regarding SPG302’s neuroprotective effects in experimental glaucoma.

Lastly, though not evaluated in the current study, the potential efficacy of SPG302 in ameliorating glaucoma-associated synaptic changes beyond the retina should be considered. Degeneration of synaptic inputs to the lateral geniculate and visual cortex has been described in rodent and primate glaucoma models (Yucel and Gupta, 2008, 2015), and atrophy of visual areas is evident in MRI imaging of human glaucoma patients (Yucel et al., 2001). These and other observations redefine glaucoma as a broader neurodegenerative disease (Yucel and Gupta, 2008, 2015; Yucel et al., 2001) and raise the prospect that a synaptic regenerative therapy may improve visual function from transduction to processing at multiple steps in the visual circuit.

## 5. Conclusion

In summary, SPG302 demonstrates therapeutic potential for protecting RGCs and restoring vision through the modulation of synaptic activity in glaucomatous neurodegeneration. Further research is needed to elucidate the mechanisms by which SPG302 safeguards and preserves the structural and functional integrity of retinal synapses and dendrites against glaucomatous insults, including elevated IOP and oxidative stress.

## Supplementary Material

1

## Data Availability

The data presented in this study are available on request from the corresponding author.

## Appendix A. Supplementary data

Supplementary data to this article can be found online at https://doi.org/10.1016/j.exer.2025.110640.
