# Supplementary material for: SPG302 protects retinal ganglion cells and preserves visual function by preserving synaptic activity in a mouse model of glaucoma
Source: Exp Eye Res. Author manuscript; Available in PMC 2026 Jun 23. (PMC13290018; doi:10.1016/j.exer.2025.110640)
Supplement: 1 [file NIHMS2182870-supplement-1.docx]

**Supplementary Table 1**. List of antibodies

| Target antigen | Vendor or Source | Catalog No. | Working dilution |
| --- | --- | --- | --- |
| Bassoon | Abcam | Ab82958 | 1:100 |
| NF68 | Millipore Sigma | N5139 | 1:500 |
| PSD95 | Abcam | Ab18258 | 1:100 |
| RBPMS | Novus Biologicals | NBP2-20112 | 1:500 |
| Synaptophysin | GeneTex | GTX633821 | 1:300 |
| TUJ1 | BioLegend | 801202 | 1:500 |
| Alexa Fluor-488 conjugated donkey  anti-rabbit IgG antibody | Invitrogen | A-21206 | 1:100 |
| Alexa Fluor-568 conjugated donkey  anti-mouse IgG antibody | Invitrogen | A-10037 | 1:100 |
| Alexa Fluor-647 conjugated donkey  anti-chicken IgG antibody | Jackson ImmunoResearch | 703-605-155 | 1:100 |

**Supplementary Table 2**. Effect of SPG302 administration on RBPMS-positive RGC survival in the middle and peripheral retina from glaucomatous mice induced by MB-induced ocular hypertension, related to Figure 2.

| Group | Age  (Months) | RGC density per retina  (RGCs/mm^2^) | |
| --- | --- | --- | --- |
|  | | Middle | Peripheral |
| DMSO-CNT | 5 | 3734 ± 132 | 3005 ± 86 |
| DMSO-MB | 5 | 3168 ± 84 | 2424 ± 76 |
| SPG302-MB | 5 | 3506 ± 59 | 2837 ± 55 |
| SPG302-CNT | 5 | 3471 ± 42 | 2825 ± 63 |

All results were reported as means ± SEM.

**Supplementary Table 3**. Effect of SPG302 administration on RGC axons in the ONHs from glaucomatous mice induced by MB-induced ocular hypertension, related to Figure 4.

| Group | Age  (Months) | Number of RGC axons (μm^2^) |
| --- | --- | --- |
| DMSO-CNT | 5 | 0.83 ± 0.02 |
| DMSO-MB | 5 | 0.70 ± 0.02 |
| SPG302-MB | 5 | 0.81 ± 0.03 |
| SPG302-CNT | 5 | 0.81 ± 0.02 |

All results were reported as means ± SEM.

**Supplementary Table 4**. Effect of SPG302 administration on pERG amplitude and pVEP amplitude and latency from glaucomatous mice induced by MB-induced ocular hypertension, related to Figure 5.

| Group | Age (Months) | Visual function tests (pERG and pVEP) |
| --- | --- | --- |
|  | | pERG Amplitude |
| DMSO-CNT | 5 | 42.89 ± 1.20 |
| DMSO-MB | 5 | 8.52 ± 1.23 |
| SPG302-MB | 5 | 17.83 ± 2.05 |
| SPG302-CNT | 5 | 42.14 ± 1.41 |
|  | | pVEP Amplitude |
| DMSO-CNT | 5 | 37.26 ± 3.23 |
| DMSO-MB | 5 | 26.91 ± 1.30 |
| SPG302-MB | 5 | 30.46 ± 2.69 |
| SPG302-CNT | 5 | 40.61 ± 2.79 |
|  | | pVEP Latency |
| DMSO-CNT | 5 | 129.8 ± 6.51 |
| DMSO-MB | 5 | 159.6 ± 7.97 |
| SPG302-MB | 5 | 158.3 ± 8.46 |
| SPG302-CNT | 5 | 135.2 ± 7.07 |

All results were reported as means ± SEM.
